# Pure and mixed clear cell carcinoma of the endometrium: A molecular and immunohistochemical analysis study

**DOI:** 10.1002/cam4.5937

**Published:** 2023-04-20

**Authors:** Casper Reijnen, Stéphanie W. Vrede, Astrid Eijkelenboom, Ruud Draak, Sanne Sweegers, Marc P. L. M. Snijders, Puck van Gestel, Johanna M. A. Pijnenborg, Johan Bulten, Heidi V. N. Küsters‐Vandevelde

**Affiliations:** ^1^ Department of Radiation Oncology Radboud University Medical Center Nijmegen The Netherlands; ^2^ Department of Obstetrics and Gynaecology Canisius‐Wilhelmina Hospital Nijmegen The Netherlands; ^3^ Department of Obstetrics and Gynaecology Radboud University Medical Center Nijmegen The Netherlands; ^4^ Department of Pathology Radboud University Medical Center Nijmegen The Netherlands; ^5^ Department of Pathology Canisius‐Wilhelmina Hospital Nijmegen The Netherlands

**Keywords:** clear cell carcinoma, endometrial cancer, molecular classification

## Abstract

**Background:**

Uterine clear cell carcinoma (CCC) consists of either pure clear cell histology but can also display other histological components (mixed uterine CCCs). In this study, the molecular and immunohistochemical background of pure and mixed uterine CCC was compared. Secondly, it was evaluated whether histological classification and molecular background affected clinical outcome.

**Methods:**

A retrospective multicenter study was performed comparing pure uterine CCCs (*n* = 22) and mixed uterine CCCs (*n* = 21). Targeted next‐generation sequencing using a 12‐gene targeted panel classified cases as polymerase‐ε (*POLE*) mutated, microsatellite instable (MSI), *TP53* wildtype or *TP53* mutated. Immunohistochemistry was performed for estrogen receptor, progesterone receptor, L1 cell adhesion molecule, MSH6, and PMS2.

**Results:**

The following molecular subgroups were identified for pure and mixed uterine CCCs, respectively: *POLE* mutated 0% (0/18) and 6% (1/18); MSI in 6% (1/18) and 50% (9/18); *TP53* wildtype in 56% (10/18) and 22% (4/18); *TP53* mutated in 39% (7/18) and 22% (4/18) (*p* = 0.013). Patients with mixed CCCs had improved outcome compared to patients with pure CCCs. Frequent *TP53* mutations were found in pure CCCs and frequent MSI in mixed CCCs, associated with clinical outcome.

**Conclusion:**

Pure and mixed uterine CCCs are two entities with different clinical outcomes, which could be explained by different molecular backgrounds. These results underline the relevance of both morphological and molecular evaluation, and may assist in tailoring treatment.

## 1 INTRODUCTION

Endometrial carcinoma (EC) is the fourth leading cancer in female patients in Europe, with 121,600 new cases and 29,600 deaths in 2018.^1^ The most common histological type of EC is endometrioid endometrial carcinoma (EEC), which accounts for 80% of all cases and generally shows a favorable prognosis.^2^ Less than 5% of EC consists of uterine clear cell carcinoma (CCC), an aggressive subtype of non‐EEC.^3^, ^4^, ^5^ Uterine CCC is frequently diagnosed in older, postmenopausal women, with 40%–45% presenting with extra‐uterine disease.^6^, ^7^ The 5‐year overall survival (OS) is 63%, compared to 83% in the EEC population.^8^ The Society of Gynecologic Oncology recommends comprehensive surgical staging and the use of adjuvant radiotherapy and/or chemotherapy for patients with uterine CCC, given the high incidence of recurrence.^9^, ^10^

In addition to pure clear cell histology, it is not uncommon for uterine CCC to display other histological components. These so‐called mixed uterine CCCs usually show an additional component of endometrioid or serous carcinoma.^11^ In serous EC, it is known that carcinomas with mixed histology have a significantly better prognosis than patients with serous histology only.^12^ Whether mixed uterine CCCs display a better clinical outcome than pure uterine CCCs remains unclear.

Major advances in characterization of the molecular background of ECs have been made in recent years.^13^ The Cancer Genome Atlas (TCGA) has defined four distinct molecular subtypes each with prognostic relevance. These molecular subtypes have been modified for clinical use by, among others, the Proactive Molecular Risk Classifier for Endometrial Cancer (ProMisE) classification.^14^, ^15^ Molecular profiling has demonstrated to be supportive in high grade EC, yet for CCC, data are limited. Molecular subtypes in EC include an “ultramutated” subgroup with mutations in the exonuclease domain of polymerase‐ε (*POLE*) and an excellent prognosis; a “hypermutated” subgroup with microsatellite instability (MSI); a “copy‐number high” subgroup characterized by *TP53* mutations and generally unfavorable outcome; and the copy number‐low subgroup.^13^ Recent studies have shown that pure uterine CCC is a molecularly heterogeneous disease which encompasses different molecular subtypes.^16^, ^17^, ^18^, ^19^, ^20^ Due to this heterogeneous molecular background, clinical behavior and prognosis of uterine CCC may be more varied than generally thought, which could have consequences for the extent of (adjuvant) therapy.

The primary aim of this study was to identify and compare the molecular and immunohistochemical (IHC) background of pure and mixed uterine CCC. The secondary aim was to evaluate whether histological classification, molecular and IHC features affect clinical outcome.

## 2 MATERIALS AND METHODS

### 2.1 Patient cohort

The nationwide Netherlands database of histopathology and cytopathology (PALGA) was used to search for all patients diagnosed with uterine CCC between January 1990 and December 2020 at the Radboud University Medical Center and the Canisius Wilhelmina Hospital Nijmegen, The Netherlands.^21^ Patients were excluded when having less than 10% clear cell component, when not receiving a surgical treatment and when no histological tumor tissue could be retrieved for IHC/molecular analysis.

### 2.2 Data collection

Clinicopathological data were collected regarding age at diagnosis, body mass index, cancer antigen‐125, cervical cytology, preoperative endometrial sampling, extent of primary surgical approach, stage, adjuvant treatment, and follow‐up data. Stage of disease was based on the 2009 International Federation of Gynecology and Obstetrics (FIGO) endometrial cancer criteria.^22^

### 2.3 Histopathological review

Hematoxylin and eosin (H&E) slides of the hysterectomy specimens were systematically reviewed by two pathologists with special interest in gynecology (J.B., H.K.), being blinded to any clinical or histological data. Histological review included classification of tumor histology, an estimation of percentages of the different components if present, depth of myometrial invasion and the presence of cervical stromal invasion (CI). Slides were screened for the presence of lymphovascular space invasion. Diagnosis was made according to the 2020 World Health Organization (WHO) guidelines and a tumor was classified as mixed when it contained at least two different histological components, regardless of component percentage.^11^ To support the diagnosis mixed uterine CCC, IHC stains were used in doubtful cases according to the 2020 WHO guidelines. For each case, an H&E slide with representative tumor tissue was selected and marked off for the purpose of DNA extraction in parallel unstained slides. In case of mixed uterine CCC, the different components were marked off separately, if possible.

### 2.4 IHC staining

IHC staining was performed for estrogen receptor (ER), progesterone receptor (PR), L1 cell adhesion molecule (L1CAM), PMS2, and MSH6 (Appendix A). For ER and PR, the number of stained tumor nuclei was scored. Cases were dichotomized, using 10% as a cutoff value. For L1CAM, the number of tumor cells showing membranous expression was scored and dichotomized, using 10% as a cutoff value. Mismatch repair deficiency (MMRd) was defined as total loss of nuclear staining of either PMS2 or MSH6, in the presence of a positive internal control.

### 2.5 DNA extraction and library preparation

Representative tumor tissue was selected by means of microdissection from 8 × 10 μm thick formalin‐fixed, paraffin‐embedded (FFPE) sections. In case of mixed histology, both components were microdissected separately. Next, tissue was digested at 56°C for at least 16 h in the presence of TET‐lysis buffer (1 M Tris/HCL pH 8.5, 0.5 M EDTA pH 8.0, 20% Tween‐20) with 5% Chelex‐100 (Bio‐Rad) and 10% proteinase K (Qiagen), followed by inactivation at 95°C for 10 min. Twice, the supernatant was transferred to a clean tube after centrifugation at 14,000 × ***g*** for 10 min. DNA concentration was determined using the Qubit 1× dsDNA High Sensitivity Assay Kit (Thermo Fisher Scientific). The isolated DNA was stored at −20°C.

The samples were analyzed with single‐molecule molecular inversion probes (smMIPs, Integrated DNA Technologies).^23^ The panel consisted of 12 relevant genes involved in EC oncogenesis as well as a number of genes informative for ProMisE classification (*AKT1*, *ARID1A*, *CTNNB1*, *ERBB2*, *FGFR2*, *KRAS*, *MTOR*, *NRAS*, *PIK3CA*, *PTEN*, *POLE*, *TP53*, Appendix B), in addition to markers for microsatellite instability (MSI). Targeted sequencing with smMIPs was performed as previously described.^23^ All smMIPs were designed in a tiling manner for hotspots in oncogenes and all coding as well as splice site consensus sequences of tumor suppressor genes (TSGs), with preferential targeting of both strands by two smMIPs. The smMIP probes were constructed by an extension and ligation probe arm (40 bp long) with a 112 bp gap and a common backbone sequence for PCR‐based library amplification. The ligation probe arm and backbone were connected by means of an 8 bp degenerate sequence (8xN) serving as a Unique Molecular Identifier (“single molecule tag”). The smMIP probes were mixed and phosporylated with 1 μL of T4 polynucleotide kinase (M0201; New England Biolabs) per 25 μL of 100 μmol/L smMIPs and ATP‐containing G4 DNA ligase buffer (B0202, New England Biolabs). The molecular ratio between gDNA and smMIPs was set at 1:3200 for each individual smMIP, and the standard genomic DNA input was 100 ng. Next, a capture mix was made (volume: 25 μL) with the phosporylated smMIP pool, 1 unit of Ampligase DNA ligase (A0110K; EpiBio) and Ampligase Buffer (A1905B, DNA ligase buffer), 3.2 units of Hemo Klentaq (M0332; New England Biolabs), 8 mmol of dNTPs (28‐4065‐20/‐12/‐22/‐32; GE Healthcare), and 100 ng of genomic DNA in a 20 μL volume. This capture mix was denatured at 95°C for 10 min and subsequently incubated for probe hybridization, extension and ligation at 60°C for 18 h. After cooling, to perform exonuclease treatment, Exonuclease I (10 units; M0293; New England Biolabs) and III (50 units; M0206; New England Biolabs) and Ampligase Buffer was added to the capture mix (total of 27 μL). The mix was incubated at 37°C for 45 min, with subsequent inactivation at 95°C for 2 min. Twenty microliters was then used for PCR in a total volume of 50 μL including a common forward primer, bar‐coded reverse primers, and iProof high fidelity master mix (1725310, Bio‐Rad). The resulting PCR products were pooled and purified with 0.8× volume of Agencourt Ampure XP Beads (A63881, Beckman Coulter).

### 2.6 Sequencing and analysis

The purified libraries were sequenced on a NexSeq500 instrument (Illumina). The Sequence Pilot software (version 4.4.0; JSI Medical Systems) was used to demultiplex the bar‐coded reads and create consensus (“unique”) reads to minimize sequencing errors. Variant calling was performed and variants were annotated as benign, likely benign, unknown, likely pathogenic or pathogenic using publicly available databases such as The Clinical Knowledgebase (https://www.jax.org/clinical‐genomics/ckb), ClinVar (https://www.ncbi.nlm.nih.gov/clinvar/), Cancer Genome Interpreter (https://www.cancergenomeinterpreter.org/home), and the Catalog of Somatic Mutations in Cancer (cancer.sanger.ac.uk/cosmic).^24^ The last three categories were considered relevant and consisted of known activating hotspot mutations for the oncogenes,^25^ and frameshift, nonsense, missense, and splice‐site mutations for the TSGs. The following molecular subgroups were identified based on these sequencing results: *POLE* mutated, MSI, *TP53* wildtype and *TP53* mutated. In case of double classifiers (for example, *POLE* and *TP53* mutation), the tumor was classified as previously described. It was previously shown that there is an excellent correlation between *TP53* mutational status and p53 IHC, and we therefore decided not to perform additional IHC in this study.^26^

### 2.7 Statistical analysis

For statistical analyses, Statistical Package for the Social Sciences, version 25.0 (IBM) was used. Clinicopathological differences between subgroups were compared using the Fisher's exact test and chi‐square for discrete variables and the Mann–Whitney *U*‐test for continuous variables. Survival analyses were performed using the Kaplan–Meier (KM) curves and univariable and multivariable Cox‐regression analysis. A recurrence was defined as first sign of relapse after a 6‐month disease‐free interval after initial surgery. Disease‐free survival was calculated from the date of initial surgery until the date of recurrence, whereas OS was calculated from the date of initial surgery until the date of death or, for surviving patients, to the date of last follow‐up. Disease‐specific survival (DSS) was calculated from the date of primary treatment to the date of death caused by the disease or, for surviving patients, to the date of the last follow‐up.

### 2.8 Ethics considerations

For this observational study, the Research Ethics Committee of the Radboud University Medical Center declared the study protocol in accordance with the applicable rules concerning the review of research ethics committees and informed consent (approval number 2018‐4023).

## 3 RESULTS

### 3.1 Patients

A total of 72 patients were identified of which 29 were excluded (*n* = 8 after pathology review, *n* = 13 due to insufficient tissue, *n* = 2 because of missing follow‐up, and *n* = 6 due to palliative treatment). A total of 43 patients were included in the analysis, of which 22 (51%) were pure uterine CCC and 21 (49%) mixed uterine CCC. Median age was 70 years (range 48–88) and did not differ between patients with pure or mixed uterine CCC (Table 1). The second histological component in mixed uterine CCC consisted of endometrioid histology in 16 patients (76%), serous histology in 3 patients (14%), and endometrioid + serous histology in 2 patients (10%). In patients with pure uterine CCC, 12 patients (55%) presented with FIGO Stages III and IV disease, compared to 6 patients (29%, *p* = 0.084) with mixed uterine CCC. As shown in Table 2, ER, PR, and IHC stains were reflective of the mixed components as the clear cell component was mostly negative for ER and PR and the other histological components were mostly positive. The use of adjuvant therapy did not differ significantly between groups. In patients with pure uterine CCC, 11 patients (50%) died in the follow‐up period, compared to 4 patients (19%, *p* = 0.033) with mixed uterine CCC.

**TABLE 1:** Baseline characteristics.

	All (*n* = 43)	Pure (*n* = 22)	Mixed (*n* = 21)	*p*‐Value
Age (years)	70 (49–88)	71 (49–84)	68 (51–88)	0.780
BMI (kg/m^2^)	27 (19–50)	26 (19–40)	28 (20–50)	0.621
CA‐125 at diagnosis (IU/mL)	19 (2–508)	22 (9–508)	16 (2–175)	0.259
Follow‐up (months)	34 (3–194)	22 (3–168)	41 (3–194)	0.174
Second histological component				
Endometrioid			16 (76)	
Serous			3 (14)	
Endometrioid + serous			2 (10)	
Myometrial invasion				0.393
<50%	18 (42)	8 (36)	10 (48)	
>50%	24 (56)	14 (64)	10 (48)	
Unknown	1 (2)	0	1 (5)	
Cervical stroma invasion				0.162
Present	18 (42)	12 (55)	6 (29)	
Not present	24 (56)	10 (46)	14 (67)	
Unknown	1 (2)	0	1 (5)	
Lymph nodes				0.435
Negative	17 (40)	8 (36)	9 (43)	
Positive pelvic nodes	4 (9)	3 (14)	1 (5)	
Positive para‐aortal nodes	9 (21)	6 (27)	3 (14)	
Unknown	13 (30)	5 (23)	8 (38)	
International Federation of Gynecology and Obstetrics (FIGO) stage				
Stages I and II	25 (58)	10 (46)	15 (71)	0.084
Stages III and IV	18 (42)	12 (55)	6 (29)	
Adjuvant therapy				
Radiotherapy	21 (51)	10 (46)	11 (52)	0.932
Chemotherapy	5 (12)	3 (14)	2 (10)	
Chemoradiotherapy	2 (5)	1 (5)	1 (5)	
None	14 (33)	8 (36)	6 (29)	
Unknown	1 (2)		1 (5)	
Residual disease				
Yes	9 (21)	7 (32)	2 (10)	0.072
No	34 (79)	15 (68)	19 (91)	
Recurrence^a^				
Yes	6 (18)	4 (27)	2 (11)	0.095
No	28 (82)	11 (73)	17 (90)	
Deceased				
Yes	15 (35)	11 (50)	4 (19)	0.033
No	28 (65)	11 (50)	17 (81)	
Deceased EC‐related				0.065
Yes	14 (33)	10 (46)	4 (19)	
No	29 (67)	12 (55)	17 (81)	

*Note*: *p*‐Values were obtained using the Mann–Whitney *U*‐test, Fisher's exact test, and chi‐squared test. Values are presented as median (range) or number (%).

Abbreviations: BMI, body mass index; CA‐125, cancer antigen‐125; EC, endometrial cancer.

^a^ Excluding patients with residual disease.

**TABLE 2:** Immunohistochemical staining.

	Pure	Mixed	
		Clear cell component	Other component
Estrogen receptor			
Positive	5 (24)	11 (52)	21 (100)
Negative	16 (76)	10 (48)	0
Progesterone receptor			
Positive	1 (5)	6 (29)	16 (84)
Negative	21 (96)	15 (71)	3 (16)^a^
L1CAM			
Negative	5 (23)	6 (29)	16 (76)
Positive	17 (77)	15 (71)	5 (24)
PMS2			
Positive	22 (100)	20 (95)	20 (95)
Negative	0	1 (5)	1 (5)
MSH6			
Positive	21 (95)	14 (67)	14 (67)
Negative	1 (5)	7 (33)	7 (33)

Abbreviation: L1CAM, L1 cell adhesion molecule.

^a^ Not assessable in two cases.

### 3.2 Molecular patterns

Of 43 patients, 8 were excluded for analyses due to either poor quality DNA and/or failed sequencing, leaving 35 patients for analysis. In one patient with mixed uterine CCC/serous carcinoma, a *POLE* hotspot mutation (c.857C>G) was found (both components could not be separately extracted, Table 3). Within the pure uterine CCCs, one patient had a tumor with MSI (5%), while nine patients (43%) with mixed uterine CCC (*p* = 0.004) showed MSI. In seven patients (41%) with pure uterine CCC, a *TP53* mutation was found, which was also the case for the mixed uterine CCCs (39%). Within the *TP53* mutated cases, MSI was present in two tumors (both mixed uterine CCCs). The single *POLE* mutated (mixed) uterine CCC in addition harbored a *TP53* mutation. Other mutation frequencies did not differ significantly between pure and mixed CCCs (Table 3).

**TABLE 3:** Molecular patterns of pure and mixed clear cell carcinomas.

	Pure CC	Mixed	*p*‐Value
*AKT*			1.000
Wildtype	16 (94)	17 (94)	
Mutated	1 (6)	1 (6)	
*ARID1A*			0.318
Wildtype	10 (59)	7 (39)	
Mutated	7 (41)	11 (61)	
*CTNNB1*			1.000
Wildtype	16 (94)	17 (94)	
Mutated	1 (6)	1 (6)	
*ERBB2*			1.000
Wildtype	15 (88)	16 (89)	
Mutated	2 (12)	2 (11)	
*KRAS*			
Wildtype	17 (100)	16 (89)	0.486
Mutated	0	2 (11)	
*MTOR*			0.229
Wildtype	17 (100)	15 (83)	
Mutated	0	3 (17)	
*NRAS*			1.000
Wildtype	16 (94)	17 (94)	
Mutated	1 (6)	1 (6)	
*PIK3CA*			0.289
Wildtype	13 (77)	10 (56)	
Mutated	4 (24)	8 (44)	
Polymerase‐ε (*POLE*)			1.000
Wildtype	17 (100)	17 (94)	
Mutated	0	1 (6)	
*PTEN*			
Wildtype	14 (82)	9 (50)	0.075
Mutated	3 (18)	9 (50)	
*TP53*			1.000
Wildtype	10 (59)	11 (61)	
Mutated	7 (41)	7 (39)	
*MSI*			0.004
No	21 (96)	12 (57)	
Yes	1 (5)	9 (43)	
Molecular subgroup			0.013
*POLE* mutated	0	1 (6)	
MSI	1 (6)	9 (50)	
*TP53* wildtype	10 (56)	4 (22)	
*TP53* mutated	7 (39)	4 (22)	

Within the whole study group, the following molecular subgroups were identified: *POLE* mutated in 1 patient (3%); MSI in 10 patients (28%); *TP53* wildtype in 14 patients (39%); and *TP53* mutated in 11 patients (31%). Analyzing pure and mixed uterine CCCs separately, the *POLE* mutated subgroup was found in respectively 0% and 6% of patients; the MSI subgroup in 6% and 50%; the *TP53* wildtype subgroup in 56% and 22%; and the *TP53* mutated subgroup in 39% and 22%, respectively (*p* = 0.013, Table 3).

**FIGURE 1:**
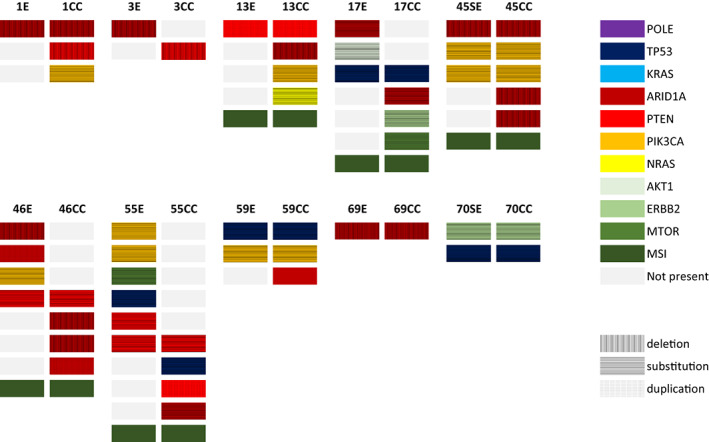
Display of all next‐generation sequencing derived mutated genes in 10 cases with mixed uterine clear cell carcinomas in which both component were sequenced separately. The colors indicate specific genes (see legend). CC, clear cell; E, endometrioid; SE, serous. MSI, microsatellite instable.

In 10 patients with mixed uterine CCC, both components could be sequenced separately (Figure 1). In eight patients, at least one shared mutation was found (Appendix C). A total of 13 mutations were found in both components (three *ARID1A*; one *ERRB2*; three *PIK3CA*; three *PTEN*; three *TP53*). A total of 26 mutations were only found in one of the components and can be seen as unique variants (one *AKT1*; twelve *ARID1A*; one *MTOR*; one *NRAS*; five *PIK3CA*; four *PTEN*; two *TP53*). Excluding patients with *POLE* mutated and MSI tumors, six mutations were found in both components, and only four mutations were only found in one of the components.

### 3.3 Immunohistochemical staining patterns

In Table 2, IHC staining patterns are shown. The clear cell component in mixed uterine CCC was ER positive in 52% of patients, compared to 24% in pure uterine CCCs (*p* = 0.111). PR was positive in 29% of mixed uterine CCCs, compared to 5% in pure uterine CCCs (*p* = 0.046), while L1CAM was positive in 71% compared to 77% in mixed versus pure uterine CCCs, respectively (*p* = 0.736). Within the endometrioid/serous component, loss of hormone receptors and L1CAM positivity was seen less frequently: ER positivity in 100%; PR positivity in 84%; L1CAM positivity in 24%. PMS2 staining was deficient in one case. Loss of MSH6 was present in 29% of mixed CCC, compared to 5% in pure uterine CCCs (*p* = 0.046). In case of mixed uterine CCCs, MSH6 and PMS2 expression was concordant in both components. MMR IHC and MSI results were concordant in all cases, except for one patient with a mixed uterine CCC showing MSI but intact IHC expression of both MSH6 and PMS2.

### 3.4 Outcome

**FIGURE 2:**
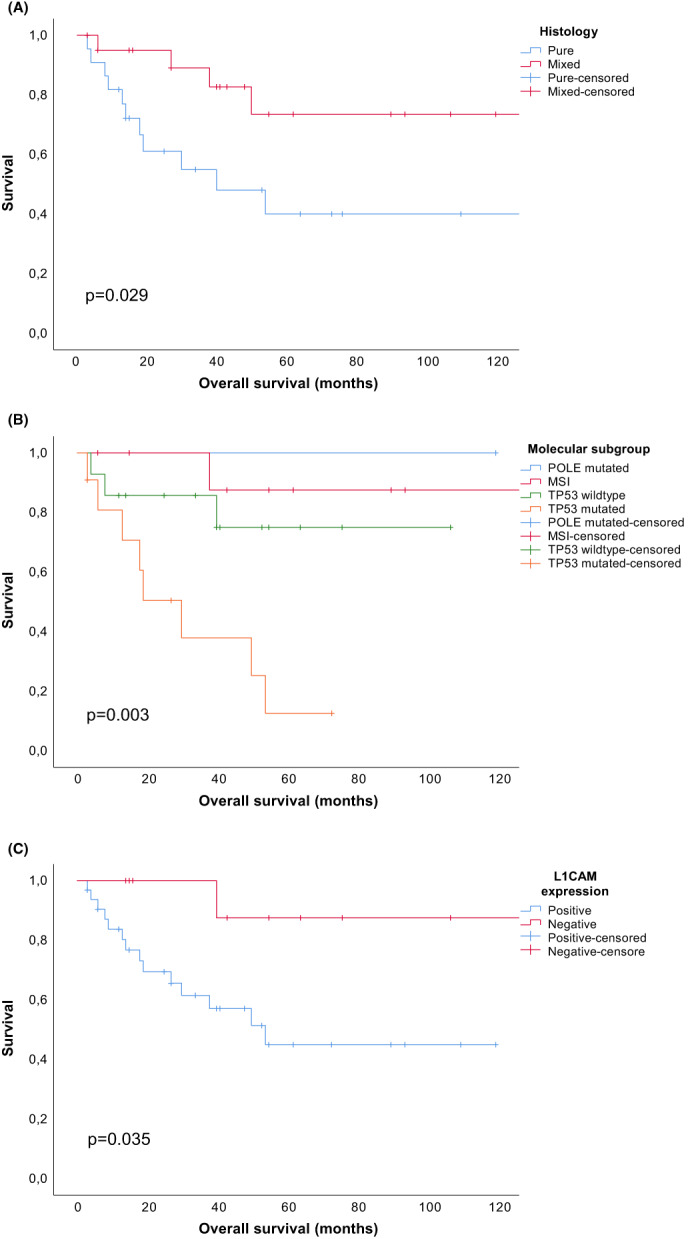
Kaplan–Meier curves displaying overall survival according to histology (A), molecular subgroup (B), and L1 cell adhesion molecule expression (C).

Figure 2A shows that patients with mixed uterine CCC had a superior OS compared to patients with pure uterine CCC (log‐rank test: *p* = 0.029), which is also the case for DSS and PFS (Appendix D, *p* = 0.045 and 0.034, respectively). As can be appreciated from Figure 2B, OS was inferior in the *TP53* mutated subgroup (log‐rank test: *p* = 0.003) whereas patients with *POLE* mutations and MSI showed very favorable outcome. DSS and PFS were inferior in the *TP53* mutated subgroup as well (Appendix D, *p* = 0.001 and 0.022). Remarkably, patients with negative L1CAM had a superior OS (*p* = 0.035, Figure 2C), as well as a superior DSS and PFS (Appendix D, *p* = 0.044 and 0.035). In univariable Cox regression analysis, histology and the molecular subgroups were correlated with OS, DSS, and PFS (Table 4). In multivariable Cox regression analysis, however, histology was not correlated with outcome, whereas molecular subgroups were correlated with OS and DSS.

**TABLE 4:** Overall survival (OS) by histology in univariable and multivariable Cox regression analysis.

	Univariable		Multivariable	
	HR (95% CI)	*p*‐Value	HR (95% CI)	*p*‐Value
*Overall survival*				
Histology				
Pure				
Mixed	0.30 (0.10–0.94)	0.04	0.69 (0.15–3.18)	0.64
Age	1.02 (0.96–1.08)	0.60	–	
International Federation of Gynecology and Obstetrics (FIGO)				
I/II				
III/IV	5.3 (1.66–16.8)	0.01	2.39 (0.62–9.19)	0.21
The Cancer Genome Atlas (TCGA)				
*TP53* wildtype				
Polymerase‐ε (*POLE*) mutated	No events	–	No events	–
MSI	0.37 (0.04–3.55)	0.39	0.61 (0.05–6.79)	0.69
*TP53* mutated	4.69 (1.24–17.8)	0.02	3.94 (1.00–15.6)	0.05
*Disease‐specific survival*				
Histology				
Pure				
Mixed	0.32 (0.10–1.04)	0.06	1.05 (0.20–5.60)	0.95
Age	1.00 (0.94–1.07)	0.91	–	
FIGO				
I/II				
III/IV	7.23 (1.99–26.3)	0.01	3.84 (0.83–17.8)	0.09
TCGA				
*TP53* wildtype				
*POLE* mutated	No events	–	No events	
MSI	0.54 (0.05–6.00)	0.62	0.93 (0.07–11.8)	0.95
*TP53* mutated	7.15 (1.51–33.9)	0.01	6.50 (1.24–34.2)	0.03
*Progression‐free survival*				
Histology				
Pure				
Mixed	0.31 (0.10–0.99)	0.05	0.59 (0.14–2.56)	0.49
Age	0.96 (0.91–1.02)	0.22	–	
FIGO				
I/II				
III/IV	8.58 (2.39–30.8)	0.01	4.92 (1.06–22.9)	0.04
TCGA				
*TP53* wildtype				
*POLE* mutated	No events	–	No events	–
MSI	0.32 (0.04–2.89)	0.31	0.98 (0.08–11.9)	0.99
*TP53* mutated	3.29 (0.95–11.5)	0.06	2.93 (0.80–10.7)	0.10

Abbreviations: CI, confidence interval; HR, hazard ratio.

## 4 DISCUSSION

The primary aim of this study was to identify and compare the molecular and IHC background of pure and mixed uterine CCC in association with clinical outcome. Interestingly, all TCGA subgroups were observed within this cohort, in line with previous findings showing that uterine CCCs are molecularly heterogeneous.^19^ Only one mixed uterine CCC with a *POLE* mutation was found with an excellent outcome. In pure uterine CCCs, no *POLE* mutations were found, in line with a previous study by Hoang et al.^19^ DeLair et al. however, comprehensively sequenced a cohort of 32 pure CCCs and did find two patients with pathogenic *POLE* mutations.^18^ Interestingly, in our study, mixed uterine CCCs were found to be MSI frequently, whereas pure uterine CCCs were mainly microsatellite stable. DeLair et al. found MMRd in 19%, whereas Hoang et al. found MSI in none of the evaluated tumors.

The secondary aim was to evaluate whether histological classification, molecular and IHC features affect clinical outcome. In KM analysis, outcome was correlated to histology, molecular subgroups, and L1CAM status. In multivariable Cox regression analysis, molecular status was correlated to OS and DSS, whereas histology was not. These results may suggest that differences in outcome between pure and mixed uterine CCCs may rather be explained by distinct molecular background. *TP53* mutations were found more often in pure uterine CCC, which could be contributive to their dismal prognosis.^19^, ^27^ *POLE* mutations and MSI, associated with improved outcome, were on the contrary observed in mixed uterine CCCs more frequently.

Previously, it was shown that mixed uterine CCCs harbor a superior prognosis compared to pure uterine CCCs.^20^ Also in serous ECs, it is known that mixed serous ECs harbor a superior prognosis compared to pure serous ECs.^12^ In this previous study, however, it was not investigated whether differences in prognosis could be explained by molecular signatures.

The oncogenesis of mixed tumors has not been fully elucidated. In 10 patients with mixed uterine CCCs, we sequenced both histological components separately and found that eight tumors harbored at least one shared mutation in both components. Also, in nine tumors “non‐shared” mutations were found, most frequently *ARID1A* mutations. Most “non‐shared” mutations were found in patients with *POLE* mutated or MSI tumors. A previous study has shown that both components in mixed CCCs harbored shared mutations, but also showed significant molecular heterogeneity and non‐shared mutations.^28^ These data are indicative that both components may evolve from a single clone but diverge along the way by obtaining new and unique mutations. *POLE* mutated and MSI tumors are considered as ultra/hypermutated tumors due to the acquirement of an extremely high burden of secondary mutations due to deficient DNA repair mechanisms.^13^ Even though the number of patients was limited, the observation that *POLE* mutations and MSI were seen almost exclusively in mixed tumors could indicate that these tumors actually have a high burden of secondary acquired (shared and non‐shared) mutations that lead to morphological divergence, dedifferentiation and the presence of different histological components.

A recent meta‐analysis included 136 uterine CCCs (114 pure and 22 mixed) and found similar rates of molecular subgroups: 4% *POLE* mutated, 11% MSI, 50% *TP53* wildtype, and 35% *TP53* mutated in pure CCCs.^29^ In mixed CCCs, no *POLE* mutations were found, whereas 59% was MSI, and only 18% was *TP53* mutated. In our study, similar rates of *TP53* mutated tumors were found (39% and 22%, respectively), as well as frequent MSI in mixed uterine CCCs (50%). This meta‐analysis also showed a favorable outcome in *POLE* mutated and MSI tumors, which supports recent recommendations by the European Society of Gynecological Oncology, European Society for Radiotherapy and Oncology and the European Society of Pathology, encouraging molecular classification in all ECs, especially high‐grade tumors.^10^ In the present study, we have analyzed IHC patterns within both components. In case of loss of one of the MMR proteins, absence of the protein was always seen in both components. In contrast, loss of ER and/or PR, and L1CAM positivity was discrepant in most cases, and was seen more often in the CCC component. These findings suggest that loss of hormone receptors as well as L1CAM expression is obtained in a more advanced stage within tumor progression. Compared to literature, showing ER expression in 0%–16% of uterine CCC cases, we found a somewhat higher prevalence (24%) in pure uterine CCCs, even though ER was only focally positive in 4/5 cases.^17^, ^30^, ^31^ PR expression was found in only 1 patient (5%), which is in line with literature. In mixed uterine CCCs, frequency of ER and PR expression was surprisingly high in the clear cell components of mixed uterine CCCs (52% and 29%). Previous papers have shown that mixed CCCs can display unexpected IHC staining patterns, including (patchy) ER/PR expression, which may be attributed to the fact that these tumors arise from a single clone and subsequently diverge.^28^, ^32^

As a potential target for HER2 directed antibody therapy, *ERBB2* mutations could be of interested for uterine CCCs. A pathogenic *ERBB2* mutation was found in four cases (12%), which is in line with literature showing ERBB2 mutations in 11% of patients.^18^

In our study, L1CAM expression was frequent and associated with impaired survival. Two previous studies did not find a correlation between L1CAM expression and impaired survival, possibly due to a limited sample size.^16^, ^33^ L1CAM is a transmembrane protein that is involved in increasing invasiveness, motility and metastatic potential, and has been found to be a poor prognostic factor in several cancers.^34^, ^35^ More recently, L1CAM positivity was found to be associated with resistance to platinum‐based chemotherapy in high‐risk EC.^36^ The number of CCCs in that study was limited underlining the need for studies investigating the association between L1CAM expression and therapy responsiveness within this particular subgroup.

We have performed a comprehensive molecular, IHC, and clinical analyses in a series of both pure and mixed uterine CCCs. However, there are some limitations. Due to the rare nature of these tumors, the number of patients within this series was limited. Also, because of the retrospective nature of the study and the use of FFPE tumor tissue, quality of the extracted DNA was variable, and DNA sequencing was unsuccessful in some cases. Of the 21 mixed uterine CCCs, it was possible to extract the DNA of both histological components separately in 10 cases. In the other cases, both histological components merged into one another and could not be isolated separately.

Concluding, we observed different molecular background between pure and mixed uterine CCCs. *TP53* mutations were found more frequently in patients with pure CCCs, and MSI was found more frequently in mixed uterine CCCs. An improved clinical outcome was found in patients with mixed uterine CCCs, compared to patients with pure uterine CCCs. Inferior outcome in pure CCCs may be explained by frequent *TP53* mutations, whereas superior outcome in mixed CCCs may be explained by frequent occurrence of MSI. These results underline the relevance of both morphological and molecular evaluation, and may assist in tailoring treatment.

## AUTHOR CONTRIBUTIONS

**Casper Reijnen:** Conceptualization (equal); data curation (equal); formal analysis (equal); investigation (equal); visualization (equal); writing – original draft (equal); writing – review and editing (equal). **Stéphanie W Vrede:** Data curation (equal); formal analysis (equal); investigation (equal); methodology (equal); writing – original draft (equal); writing – review and editing (equal). **Astrid Eijkelenboom:** Formal analysis (equal); investigation (equal); methodology (equal); supervision (equal); validation (equal). **Ruud Draak:** Conceptualization (equal); data curation (equal); formal analysis (equal); funding acquisition (equal); investigation (equal); writing – original draft (equal). **Sanne Sweegers:** Data curation (equal); formal analysis (equal); investigation (equal); methodology (equal); validation (equal); writing – original draft (equal); writing – review and editing (equal). **Marc PLM Snijders:** Conceptualization (equal); funding acquisition (equal); investigation (equal); methodology (equal); writing – original draft (equal); writing – review and editing (equal). **Puck van Gestel:** Conceptualization (equal); data curation (equal); formal analysis (equal); investigation (equal); methodology (equal); project administration (equal); writing – original draft (equal); writing – review and editing (equal). **Johanna MA Pijnenborg:** Conceptualization (equal); formal analysis (equal); funding acquisition (equal); investigation (equal); methodology (equal); supervision (equal); visualization (equal); writing – original draft (equal); writing – review and editing (equal). **Johan Bulten:** Conceptualization (equal); formal analysis (equal); investigation (equal); methodology (equal); resources (equal); supervision (equal); writing – original draft (equal); writing – review and editing (equal). **Heidi VN Küsters‐Vandevelde:** Conceptualization (equal); data curation (equal); formal analysis (equal); funding acquisition (equal); investigation (equal); supervision (equal); validation (equal); visualization (equal); writing – original draft (equal); writing – review and editing (equal).

## FUNDING INFORMATION

Canisius‐Wilhelmina Hospital Scientific Fund 2021.

## CONFLICT OF INTEREST STATEMENT

None declared.

## ETHICS STATEMENT

This study was performed in accordance with the Declaration of Helsinki. The study was approved by the Medical Ethics Committee of the Radboud University Medical Center (number 2018‐4023) and performed according to the Code for Proper Secondary Use of Human Tissue (Dutch Federation of Biomedical Scientific Societies, http://www.federa.org).

## CONSENT FOR PUBLICATION

Not applicable.

## Supporting information


Appendix S1

## Data Availability

The datasets used and/or analyzed during the current study are available from the corresponding author on reasonable request.
