# Supplementary material for: Pure and mixed clear cell carcinoma of the endometrium: A molecular and immunohistochemical analysis study
Source: Cancer Med. 2023 Apr 20;12(11):12365–76. doi: 10.1002/cam4.5937 (PMC10278528; doi:10.1002/cam4.5937)
Supplement: Supplementary file 1 — Appendix S1 [file CAM4-12-12365-s001.pdf]

**Appendix A. Detailed information on immunohistochemical staining.**

For ER and PR, antigen retrieval (97 $^{0}$C for 30 minutes in Tris/EDTA buffer pH 9 [Envision FLEX Target Retrieval Solution High pH, DAKO, Agilent Technologies, Santa Clara, CA, United States]) and blocking of endogenous peroxidase with hydrogen peroxide were performed. Subsequently, slides were incubated with: ER antibody (clone EP1 GA084, DAKO, Agilent Technologies, Santa Clara, CA, United States) and PR antibody (clone, Pgr 1294 GA090, DAKO, Agilent Technologies, Santa Clara, CA, United States). Envision FLEX/HRP (DAKO, Agilent Technologies, Santa Clara, CA, United States) was used and visualization was performed using Envision FLEX DAB+ Chromogen (DAKO, Agilent Technologies, Santa Clara, CA, United States).

For L1CAM, EDTA (95 $^{0}$C for 10 minutes in Tris-EDTA buffer pH 9) and blocking of endogenous peroxidase with hydrogen peroxide were performed. Subsequently, slides were incubated with: L1CAM antibody (purified anti-CD171, clone 14.10, Biolegend, San Diego, CA, US, dilution 1:100). Powervision+ Poly-HRP was used and visualization was performed using PowerVision DAB substrate solution (Leica Biosystems, Buffalo Grove, IL, US).

Immunohistochemical analysis of the mismatch repair (MMR) proteins PMS2 and MSH6 was performed. In short, blank 4µm formalin-fixed, paraffin-embedded (FFPE) sections were cut on Superfrost+ glass slides. After antigen retrieval with EnVision FLEX High pH Target Retrieval Solution, and blocking of endogenous peroxidase with hydrogen peroxide, all slides were incubated with anti-MSH6 (clone EPR3945 1:400, Abcam, Cambridge, UK) or anti-PMS2 (clone A16-4 dilution 1:20, BD Biosciences, San Jose, CA). Subsequently, they were incubated with EnVision FLEX and visualized with High pH visualization system according to the manufacturer’s instructions for use. Counterstaining was performed with hematoxylin, and the slides were dehydrated and mounted.

**Appendix B**. All gene regions targeted by the smMIP panel

| Gene | Exon | Targeted codons | Positions | RefSeq ID | Ensembl ID |
|---|---|---|---|---|---|
| *AKT1* | 3 | E17 | c.47-5 to c.86 | NM_005163 | ENST00000555528 |
| *ARID1A* | 1 to 20 | M1-Stop2286 | c.1 to c.6858 | NM_006015.5 | ENST00000324856 |
| *CTNNB1* | 3 | D32-S45 | c.53 to c.146 | NM_001904.3 | ENST00000349496 |
| *ERBB2* | 20 | Y772-Y781 | c.2308-1 to c.2357 | NM_004448 | ENST00000269571 |
| *FGFR2* | 6 to 9<br>11 to 14 | I217-V392<br>K485-L627 | c.649 to c.1174<br>c.1453 to c.1879 | NM_000141.5 | ENST00000358487 |
| *KRAS* | 2 | G12-G13 | c.9 to c.71 | NM_004985.4 | ENST00000311936 |
| | 3 | A59-Q61 | c.122 to c.215 | | |
| | 4 | K117, A146 | c.291-5 to c.357 | | |
| | | | c.402 to c.450+5 | | |
| *MTOR* | 30 | D1458-E1489 | c.4371 to c.4469+5 | NM_004958.3 | ENST00000361445 |
| | 39 | A1789-A1820 | c.5365-5 to c.5460 | | |
| | 43 | A1971-L1995 | c.5911-5 to c.5985 | | |
| | 47 | Q2194-L2220 | c.6580 to c.6662+5 | | |
| | 53 | M2404-D2433 | c.7210 to c.7300+5 | | |
| | 56 | G2484-T2509 | c.7448-5 to c.7527 | | |
| *NRAS* | 2<br>3<br>4 | G12, G13<br>A59, Q61<br>K117, A146 | c.-17-5 to c.64<br>c.161 to c.245<br>c.312 to c.450+5 | NM_002524 | ENST00000369535 |
| *PIK3CA* | 2<br>5<br>8<br>10<br>21 | S66 – I117<br>Y317 – K353<br>E418 – M441<br>D520 – H554<br>S1015 – N1068 | c.195 to c.352<br>c.947 to c.1059<br>c.1252 to c.1323<br>c.1558 to c.1664<br>c.3058 to c.3207 | NM_006218.3 | ENST00000263967 |
| *POLE* | 9 to 14 | D268-E491 | c.802-5 to c.1473+5 | NM_006231.3 | ENST00000320574 |
| *PTEN* | 1 to 9 | M1-Stop404 | c.1 to c.1210+5 | NM_000314.6 | ENST00000371953 |
| *TP53* | 2 to 11 | >95% of all coding and splice sequences (-5/+5) | c.1 to c.1180+5 | NM_000565.5 | ENST00000269305 |

## Appendix C. Sequencing results

| Study number | Variant | Variant allele frequency | *N* of mutant reads | Variant class |
|---|---|---|---|---|
| 1EEC | ARID1A:c.2178-2187del p.(Arg727fs) | 70 | 468 | 4 |
| 1CC | ARID1A:c.2178-2187del p.(Arg727fs) | 52 | 64 | 4 |
| 1CC | PIK3CA:c.3140A>G p.(His1047Arg) | 52 | 182 | 5 |
| 1CC | PTEN:c.113del p.(Pro38fs) | 33 | 68 | 4 |
| 1 | Microsatellite stable | | | |
| | | | | |
| 2 | TP53:c.396G>C p.(Lys132Asn) | 49 | 46 | 4 |
| 2 | Microsatellite stable | | | |
| | | | | |
| 3EEC | ARID1A:c.1181del p.(Pro394fs) | 47 | 14 | 4 |
| 3CC | PTEN:c.428del p.(Gly143fs) | 62 | 26 | 4 |
| 3 | Microsatellite stable | | | |
| | | | | |
| 4 | No (potentially) pathogenic variants | | | |
| | Microsatellite stable | | | |
| | | | | |
| 5 | No (potentially) pathogenic variants | | | |
| | Microsatellite stable | | | |
| | | | | |
| 6 | AKT1:c.49G>A p.(Glu17Lys) | 73 | 462 | 5 |
| 6 | ARID1A:c.6301_6302dup p.(Asp210fs) | 28 | 38 | 4 |
| 6 | CTNNB1:c.94G>T p.(Asp32Tyr) | 21 | 108 | 5 |
| 6 | TP53:c.658T>G p.(Tyr220Asp) | 70 | 142 | 4 |
| 6 | Microsatellite stable | | | |
| | | | | |
| 8 | PTEN:c.697C>T p.(Arg233*) | 58 | 1096 | 4 |
| 8 | Microsatellite stable | | | |
| | | | | |
| 10 | ARIDA1:c.1353_1354del p.(Pro452fs) | 34 | 104 | 4 |
| 10 | Microsatellite stable | | | |
| | | | | |
| 11 | No (potentially) pathogenic variants | | | |
| | Microsatellite stable | | | |
| | | | | |
| 13EEC | PTEN:c.723dup p.(Glu242*) | 30 | 92 | 4 |
| 13CC | PTEN:c.723dup p.(Glu242*) | 30 | 92 | 4 |
| 13CC | ARID1A:c.5548del p.(Asp1850fs) | 29 | 28 | 4 |
| 13CC | NRAS:c.3G>A p.(Thr2_Met67del) | 31 | 214 | 5 |
| 13CC | PIK3CA:c.3140A>G p.(His1047Arg) | 30 | 182 | 5 |
| 13 | Microsatellite instable | | | |
| | | | | |
| 15 | PTEN:c.405dup p.(Cys136fs) | 50 | 134 | 4 |

| | | | | |
|---|---|---|---|---|
| 15 | PTEN:c.635-16_636del p.(?) | 55 | 88 | 4 |
| 15 | TP53:c.537T>G p.(His179Gln) | 80 | 86 | 4 |
| 15 | Microsatellite stable | | | |
| | | | | |
| 17EEC | ARID1A:c.3219G>A p.(Try1073*) | 17 | 24 | 4 |
| 17EEC | AKT1:c.49G>A p.(Glu17Lys) | 22 | 50 | 5 |
| 17EEC | TP53:c.817C>T p.(Arg273Cys) | 61 | 289 | 4 |
| 17CC | TP53:c.817C>T p.(Arg273Cys) | 61 | 289 | 4 |
| 17CC | ARIDA1:c.3826C>T p.(Arg1276*) | 22 | 14 | 4 |
| 17CC | ERBB2:c.2524G>A p.(Val842Ile) | 39 | 142 | 5 |
| 17CC | MTOR:c.5395G>A p.(Glu1799Lys) | 41 | 966 | 5 |
| 17 | Microsatellite instable | | | |
| | | | | |
| 18 | No (potentially) pathogenic variants | | | |
| | Microsatellite stable | | | |
| | | | | |
| 19 | PIK3CA:c.1636C>A p.(Gln546Lys) | 19 | 669 | 5 |
| 19 | PIK3CA:c.1035T>A p.(Asn345Lys) | 52 | 48 | 5 |
| 19 | POLE:c.857C>G p.(Pro286Arg) | 36 | 566 | 5 |
| 19 | PTEN:c.1021T>G p.(Phe341Val) | 63 | 376 | 5 |
| 19 | MTOR:c.6644C>A p.(Ser2215Tyr) | 42 | 196 | 5 |
| 19 | MTOR:c.7513C>T p.(Arg2505*) | 28 | 248 | 5 |
| 19 | TP53:c.339C>A p.(Phe113Leu) | 16 | 381 | 5 |
| 19 | Microsatellite stable | | | |
| | | | | |
| 20 | ARID1A:c.2808del p.(Ser936fs) | 39 | 56 | 4 |
| 20 | PIK3CA:c.3140A>G p.(His1047Arg) | 21 | 22 | 5 |
| 20 | TP53:c.338T>G p.(Phe113Cys) | 16 | 43 | 5 |
| 20 | Microsatellite stable | | | |
| | | | | |
| 21 | ARID1A:c.6420del p.(Phe2141fs) | 30 | 128 | 4 |
| 21 | KRAS:c.34G>T p.(Gly12Cys) | 18 | 96 | 5 |
| 21 | PTEN:c.437dup p.(Leu146fs) | 32 | 426 | 4 |
| 21 | PIK3CA:c.278G>T p.(Arg93Leu) | 26 | 42 | 4 |
| 21 | Microsatellite instable | | | |
| | | | | |
| 27 | KRAS:c.35G>A p.(Gly12Asp) | 47 | 30 | 5 |
| 27 | PTEN:c.800del p.(Lys267fs) | 35 | 22 | 4 |
| 27 | Microsatellite instable | | | |
| | | | | |
| 29 | TP53:c.406C>T p.(Gln136*) | 5 | 10 | 4 |
| 29 | ARIDA1:c.3826C>T p.(Arg1276*) | 6,8 | 10 | 4 |
| 29 | Microsatellite stable | | | |
| | | | | |
| 31 | No (potentially) pathogenic variants | | | |
| | Microsatellite stable | | | |

| | | | | |
|---|---|---|---|---|
| 33 | ERBB2:c.2524G>A p.(Val842Ile) | 29 | 142 | 5 |
| 33 | ERBB2:c.2047C>T p.(Arg683Trp) | 5 | 10 | 5 |
| 33 | TP53:c.414del p.(Lys139fs) | 56 | 194 | 4 |
| 33 | TP53:c.1024c>T p.(Arg342*) | 22 | 202 | 4 |
| 33 | Microsatellite stable | | | |
| | | | | |
| 41 | ERBB2:c.2493+1G>A p.(?) | 5,1 | 10 | 4 |
| 41 | TP53:c.801del p.(Asn268fs) | 35 | 68 | 4 |
| 41 | TP53:c.342_344delins p.(Leu114fs) | 34 | 37 | 4 |
| 41 | Microsatellite stable | | | |
| | | | | |
| 43 | TP53:c.488A>G p.(Tyr163Cys) | 63 | 460 | 4 |
| 43 | Microsatellite stable | | | |
| | | | | |
| 44 | ARID1A:c.598C>T p.(Gln200*) | 36 | 24 | 4 |
| 44 | ARID1A:c.6092dupA p.(Tyr2031*) | 26 | 190 | 4 |
| 44 | NRAS:c.182A>G p.(Gln61Arg) | 26 | 110 | 5 |
| 44 | PIK3CA:c.1633G>A p.(Glu545Lys) | 26 | 598 | 5 |
| 44 | Microsatellite stable | | | |
| | | | | |
| 45SER | ARID1A:c.3977del p.(Pro1326fs) | 32 | 56 | 4 |
| 45SER | PIK3CA:c.1636C>G p.(Gln546Glu) | 16 | 492 | 5 |
| 45SER | PIK3CA:c.1633G>A p.(Glu545Lys) | 12 | 102 | 5 |
| 45CC | ARID1A:c.6420del p.(Phe2141fs) | 18 | 128 | 4 |
| 45CC | ARID1A:c.3977del p.(Pro1326fs) | 32 | 32 | 4 |
| 45CC | PIK3CA:c.1636C>G p.(Gln546Glu) | 14 | 492 | 5 |
| 45CC | PIK3CA:c.1633G>A p.(Glu545Lys) | 17 | 586 | 5 |
| 45CC | ARID1A:c.183del p.(Ala62fs) | 25 | 38 | 4 |
| 45 | Microsatellite instable | | | |
| | | | | |
| 46EEC | ARID1A:c.5548del p.(Asp1850fs) | 50 | 222 | 4 |
| 46EEC | ARID1A:c.3524dup p.(Leu1176fs) | 35 | 36 | 4 |
| 46EEC | PIK3CA:c.1031T>C p.(Val344Ala) | 22 | 32 | 5 |
| 46EEC | PTEN:c.71A>G p.(Asp24Gly) | 47 | 166 | 4 |
| 46CC | ARID1A:c.3216del p.(Lys1072fs) | 6,1 | 14 | 4 |
| 46CC | ARID1A:c.437del p.(Pro146fs) | 41 | 32 | 4 |
| 46CC | ARID1A:c.5548dup p.(Asp1850fs) | 33 | 12 | 4 |
| 46CC | PTEN:c.71A>G p.(Asp24Gly) | 63 | 316 | 4 |
| 46 | Microsatellite instable | | | |
| | | | | |
| 47 | TP53:c.405C>T p.(Cys135*) | 53 | 36 | 4 |
| 47 | Microsatellite stable | | | |
| | | | | |
| 55EEC | PIK3CA:c.1030G>A p.(Val344Met) | 55 | 1880 | 5 |
| 55EEC | PIK3CA:c.3139C>T p.(His1047Tyr) | 57 | 1472 | 5 |

| | | | | |
|---|---|---|---|---|
| 55EEC | MTOR:c.5395G>A p.(Glu1799Lys) | 40 | 966 | 5 |
| 55EEC | PTEN:c.697C>T p.(Arg233*) | 58 | 1096 | 4 |
| 55EEC | PTEN:c.517C>T p.(Arg173Cys) | 48 | 420 | 4 |
| 55EEC | TP53:c.1009C>T p.(Arg337Cys) | 44 | 200 | 4 |
| 55CC | TP53:c.916C>T p.(Arg306*) | 41 | 1628 | 4 |
| 55CC | PTEN:c.697C>T p.(Arg233*) | 58 | 930 | 4 |
| 55CC | PTEN:c.800dup p.(Asp268fs) | 37 | 532 | 4 |
| 55CC | ARID1A:c.3424C>T (c.Gln1142*) | 42 | 754 | 4 |
| 55 | Microsatellite instable | | | |
| | | | | |
| 58 | ARID1A:c.5693del p.(Pro1898fs) | 7,1 | 54 | 4 |
| 58 | ARID1A:c.5548del p.(Asp1850fs) | 50 | 222 | 4 |
| 58 | CTNNB1:c.121A>G p.(Thr41Ala) | 18 | 510 | 5 |
| 58 | PTEN:c.956_959del p.(Thr319fs) | 32 | 334 | 4 |
| 58 | PTEN:c.209+4_209+7del p.(?) | 29 | 634 | 5 |
| 58 | Microsatellite instable | | | |
| | | | | |
| 59EEC | PIK3CA:c.1633G>A p.(Glu545Lys) | 26 | 598 | 5 |
| 59EEC | TP53:c.743G>A p.(Arg248Gln) | 47 | 373 | 4 |
| 59CC | PIK3CA:c.1633G>A p.(Glu545Lys) | 18 | 192 | 5 |
| 59CC | TP53:c.743G>A p.(Arg248Gln) | 19 | 146 | 4 |
| 59CC | ARID1A:c.1501C.T p.(Gln501*) | 21 | 118 | 4 |
| 59 | Microsatellite stable | | | |
| | | | | |
| 66 | PIK3CA:c.1258T>C (c.Cys420Arg) | 78 | 603 | 5 |
| 66 | Microsatellite stable | | | |
| | | | | |
| 68CC | PTEN:c.1003C>T p.(Arg335*) | 51 | 108 | 5 |
| 68CC | ARID1A:c.2896G>T p.(Glu966*) | 36 | 98 | 4 |
| 68 | Microsatellite stable | | | |
| | | | | |
| 69EEC | ARID1A:c.2951del p.(Lys984fs) | 30 | 122 | 4 |
| 69CC | ARID1A:c.2951del p.(Lys984fs) | 30 | 120 | 4 |
| 69 | Microsatellite stable | | | |
| | | | | |
| 70SER | ERBB2:c.929C>T p.(Ser310Phe) | 23 | 859 | 5 |
| 70SER | TP53:c.859G>T p.(Glu287*) | 38 | 930 | 4 |
| 70CC | ERBB2:c.929C>T p.(Ser310Phe) | 13 | 254 | 5 |
| 70CC | TP53:c.859G>T p.(Glu287*) | 20 | 214 | 4 |
| 70 | Microsatellite stable | | | |
| | | | | |
| 71 | PIK3CA:c.1637A>G p.(Gln546Arg) | 21 | 1732 | 5 |
| 71 | Microsatellite stable | | | |
| | | | | |
| 72 | ARID1A:c.6420del p.(Phe2141fs) | 30 | 90 | 4 |
| 72 | ARID1A:c.5548del p.(Asp1850fs) | 36 | 28 | 4 |

| | | | | |
|---|---|---|---|---|
| 72 | PIK3CA:c.1624G>A p.(Glu542Lys) | 21 | 298 | 5 |
| 72 | Microsatellite instable | | | |

Appendix D

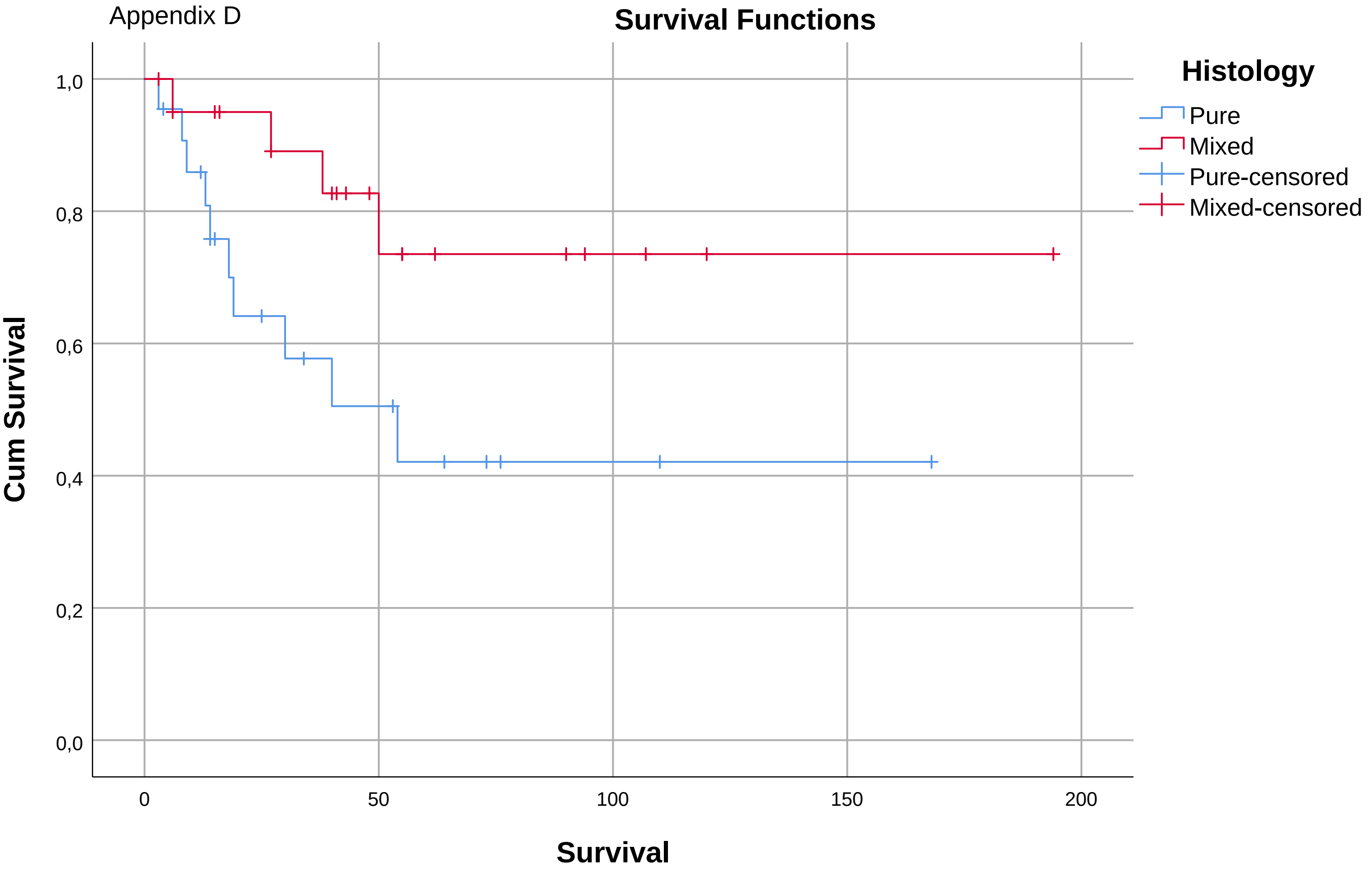

Survival Functions
Cum Survival
1,0
0,8
0,6
0,4
0,2
0,0
0
50
100
150
200
Survival
Histology
Pure
Mixed
Pure-censored
Mixed-censored

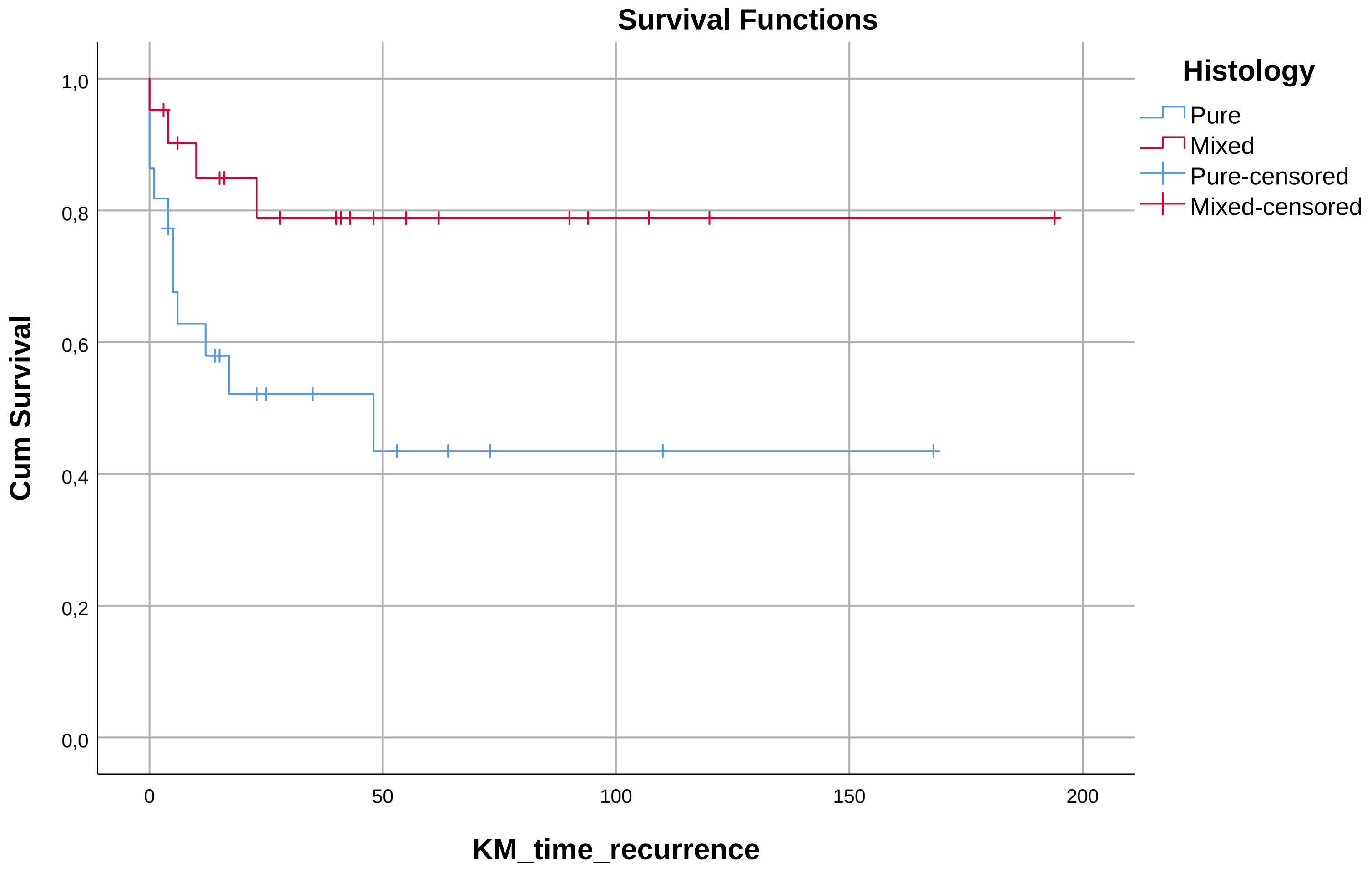

Survival Functions
Cum Survival
1,0
0,8
0,6
0,4
0,2
0,0
0
50
100
150
200
KM_time_recurrence
Histology
Pure
Mixed
Pure-censored
Mixed-censored

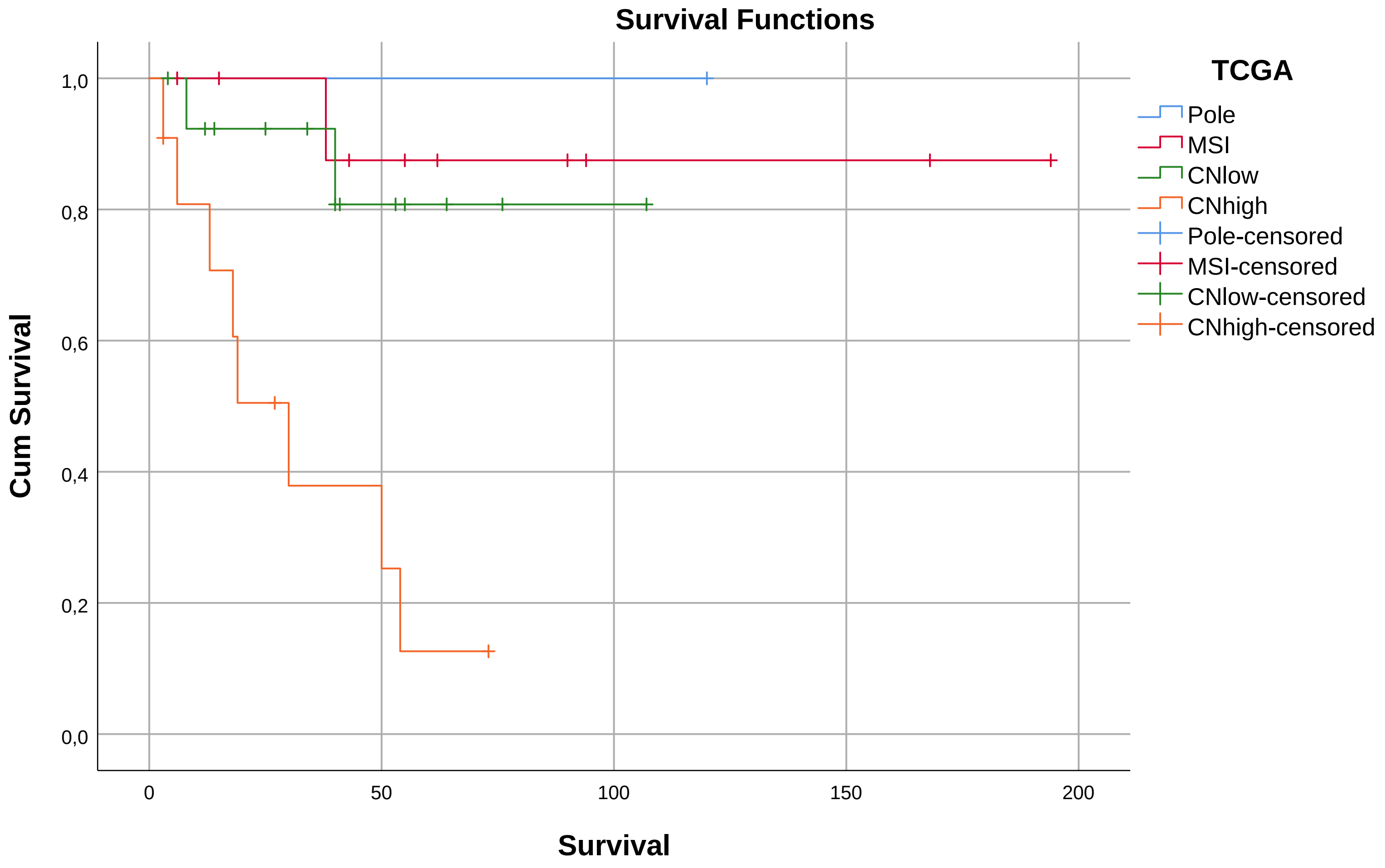

Survival Functions
TCGA
Pole
MSI
CNlow
CNhigh
Pole-censored
MSI-censored
CNlow-censored
CNhigh-censored
Cum Survival
1,0
0,8
0,6
0,4
0,2
0,0
0
50
100
150
200
Survival

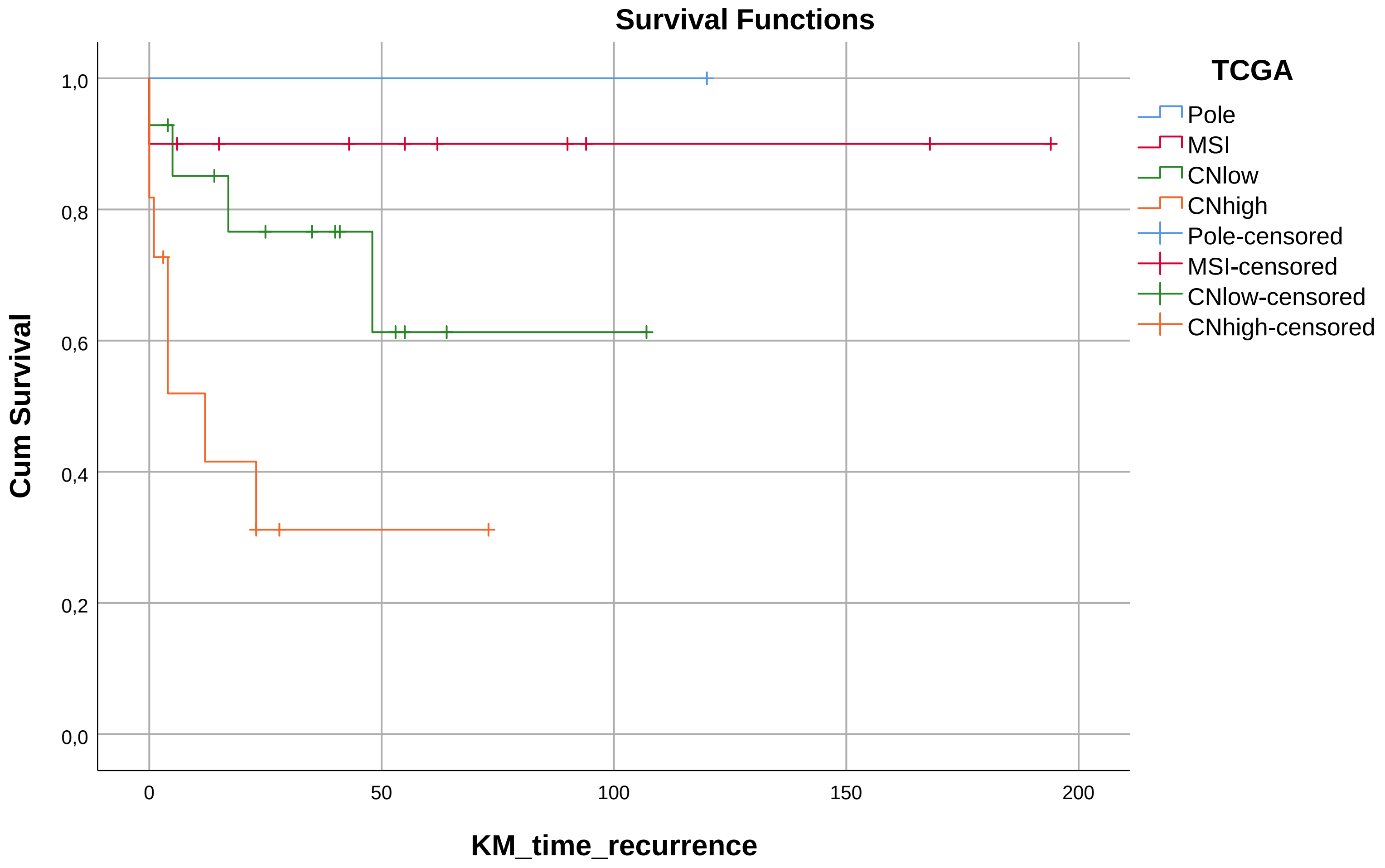

Survival Functions
Cum Survival
1,0
0,8
0,6
0,4
0,2
0,0
0
50
100
150
200
KM_time_recurrence
TCGA
Pole
MSI
CNlow
CNhigh
Pole-censored
MSI-censored
CNlow-censored
CNhigh-censored

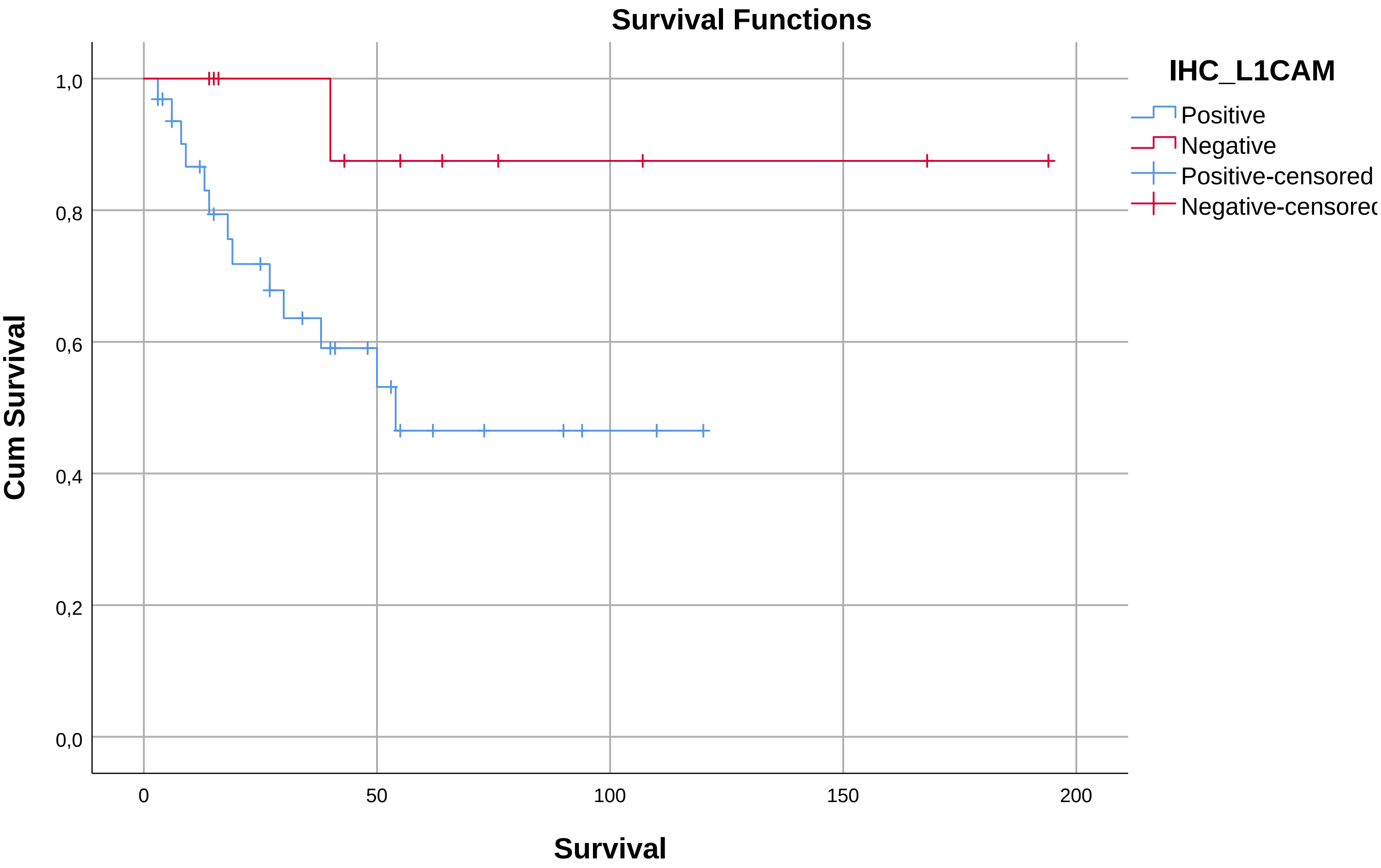

Survival Functions
IHC_L1CAM
Positive
Negative
Positive-censored
Negative-censored
Cum Survival
1,0
0,8
0,6
0,4
0,2
0,0
0
50
100
150
200
Survival

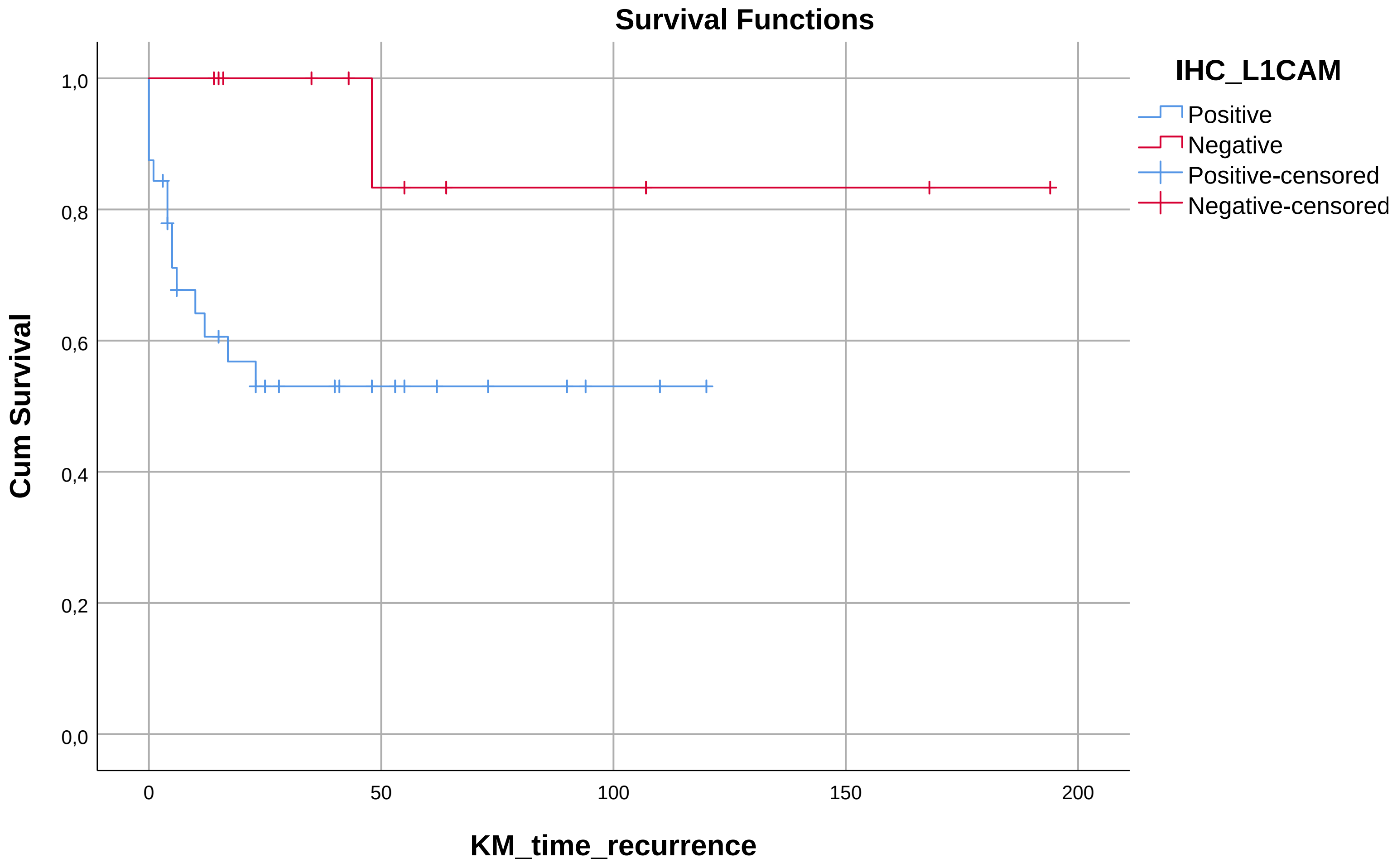

Survival Functions
Cum Survival
1,0
0,8
0,6
0,4
0,2
0,0
0
50
100
150
200
KM_time_recurrence
IHC_L1CAM
Positive
Negative
Positive-censored
Negative-censored
